# Rat Tumour Histopathology Associated with Experimental Chronic Dietary Exposure to Ochratoxin A in Prediction of the Mycotoxin’s Risk for Human Cancers

**DOI:** 10.3390/toxins13030205

**Published:** 2021-03-12

**Authors:** Diana Herman, Peter Mantle

**Affiliations:** 1 Pathology Department, County Hospital Timisoara, 300736 Timisoara, Romania; diaherman@yahoo.com; 2 Centre for Environmental Policy, Imperial College London, London SW7 2AZ, UK

**Keywords:** vimentin, CD10, MNF 116, renal cell cancer, urothelial cancer, testicular cancer, immunohistochemistry

## Abstract

Mammalian animal toxicity of ochratoxin A (OTA) has focused largely in the past half-century on pigs because of initial recognition of it as a principal cause of intermittent growth suppression and renal disease caused by mouldy feed. Subsequent classical toxicology has used laboratory rodents because renal pathology in pigs raised questions concerning possible involvement in the human idiopathic bilateral renal atrophy of Balkan endemic nephropathy for which OTA was a focus of attention for human nephropathy through 1980s and into 2000s. Emphasis on human nephropathy has more recently concerned the plant metabolite aristolochic acid. Recognition that agricultural management can often minimise food and feed-stuff spoilage by OTA-producing Aspergilli and Penicillia has moderated some of the risks for animals. Legislation for human food safety combined with sophisticated analysis generally provides safety in the developed world. Chronic experimental exposure of male rats, in the absence of clinical dis-ease, specifically causes renal cancer. The possibility of this as a unique model for the human has generated considerable experimental evidence which may be more directly relevant for carcinogenesis in the complex kidney than that obtained from biochemical toxicities in vitro. Nevertheless, there does not appear to be any case of human renal or urinary tract cancer for which there is verified etiological proof for causation by OTA, contrary to much claim in the literature. To contribute to such debate, histopathology review of OTA/rat renal cancers, augmented where appropriate by immune profiles, has been completed for all remaining tumours in our research archive. Overall consistency of positivity for vimentin, is matched with occasional positives either for CD10 or the cytokeratin MNF 116. The current situation is discussed. Suggestion that OTA could cause human testicular cancer has also been challenged as unsupported by any experimental findings in rats, where the Leydig cell tumour immune profile does not match that of human germ cell neoplasms.

## 1. Introduction

Ochratoxin A (OTA) was discovered in South Africa in the early 1960s to explain the general toxicity of cultured *Aspergillus ochraceus* as a dietary additive for experimental rats. Concurrently, the term mycotoxins became used to encompass toxic metabolites of other food-spoilage moulds such, as the aflatoxins of *A. flavus,* found to have widespread occurrence. Focus for OTA soon shifted to the spasmodic occurrence of an idiopathic porcine nephropathy in the Danish bacon industry, economically linked to home-grown barley; in some high rainfall years this had been insufficiently dried before storage. Seasonal opportunist moulding by a common *Penicillium*, and chromatographic recognition of OTA partly by its fluorescence under UV_254_ light, revealed another biosynthetic source of the mycotoxin in amounts subsequently demonstrated experimentally as a major cause of the disease in pigs. This had been expressed as reduced growth rate, low carcass weight and mottled and disproportionally enlarged kidneys, readily recognised at meat inspection and the carcass rejected [1].

Several *Aspergillus* and *Penicillium* moulds across both tropical and temperate latitudes were subsequently found to elaborate OTA during spoilage of major staple agricultural products such as cereals, and in crops for high-end commodities such as coffee, cocoa and red wines. Concern that traces of OTA in major cereal products such as pasta might pose a human health risk, and of potential economic threat to commercial images of the high-end commodities, stimulated extensive research into OTA toxicology. Concurrently, world-wide food safety authorities (e.g., for Europe, Joint expert committee on food additives, International agency for research on cancer, European food standards agency) addressed potential health risks and formulated documents to guide regulatory legislations. 

Pigs being important in the human food chain in many economies prompted a major US experimental toxicology study in the 1980s with lifetime chronic exposure in rats [2]. The striking finding of renal tumours, expressed late in life mainly in males in the absence of overt toxicity, was the more notable as designating OTA as the most potent chemical for renal carcinoma in rats, although the extensive technical report did not raise any specific concerns for humans. The kidney cancer incidence findings demonstrated across the three gavage dose rates chosen for the NTP study later demonstrated OTA as a model for classic log dose/response for chemical carcinogens [3]. 

Subsequent developments in sensitive and definitive analytical methodology have shown widespread occurrence of relevant moulds and their mycotoxins, although general occurrence in well-managed agriculture is generally low. For human health, there has been little verified etiological evidence of general toxicities attributable other than to spasmodic episodes. The initial cautious classification of possible carcinogenetic risk [4] seems never subsequently to have had evidence that could stand classical epidemiological scrutiny. Particularly, the relevance of rat and mouse as pathological models for the human has become insecure because of the wide quantitative gulf (>10^4^-fold) between necessary daily carcinogenic exposure for rodents (>30 µg OTA/kg body weight daily for at least half a rat lifetime, and much more for a mouse) and surveyed average natural daily human intake data (UK, 0.26–3.5 ng/kg b. wt.) [5].

The classical US toxicity study in rats [2] followed strict toxicology protocols involving gavage administration of OTA five days per week for up to 2 years. The more recent London studies, part of a European Commission project on OTA toxicity (2001-4), provided daily dietary ochratoxins as elaborated during moulding of shredded wheat breakfast cereal by *Aspergillus ochraceus* in a shaken solid substrate fermentation. The product, analysed for OTA, was homogenised (c.10^3^-fold) into powdered rat diet for consumption during natural diurnal habit for Fischer male rats. Although blood OTA analysis had not been available for the US study, values in London followed a gradual rise to 8–10 µg/mL during the first month. For the highest daily OTA intake employed (300 µg/kg b wt) at least 9 months exposure seemed necessary to initiate kidney cancer, during which the common prevalence of monocytic leukaemia in ageing Fischer males was apparently repressed [6]. The tumour-free outcome for the US study lowest dose (21 µg/kg b wt) was raised to c. 30 µg/kg b wt in a London study in Dark Agouti male rats [7]. The dose/response criteria adopted for OTA’s recognition as the model for log dose/response for chemical carcinogens [3] was thus reinforced by all subsequent findings in London [7] describing OTA/rat renal carcinogenicity as thresholded. Notably, this was not considered later in a major analysis of human risk assessment for OTA in Canada [8] in which a curious distorted non-thresholded graphical illustration is presented. 

Critical histology review of some OTA/rat renal tumours has been described [9], including exploratory application for the first time of clinical immunohistochemistry where most tumours showed a range of positive responses to the clinical immuno-stains. Although designed for human histopathological diagnosis some immuno-stains helpfully showed cross-reactivity to rats. The primary experimental objective here is to consolidate those findings by completing histology review of all remaining archived cases in our archive.

## 2. Results

**Figure 1:**
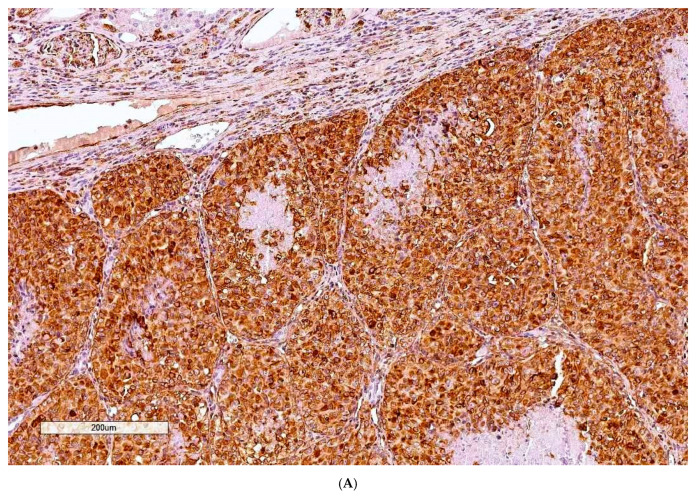

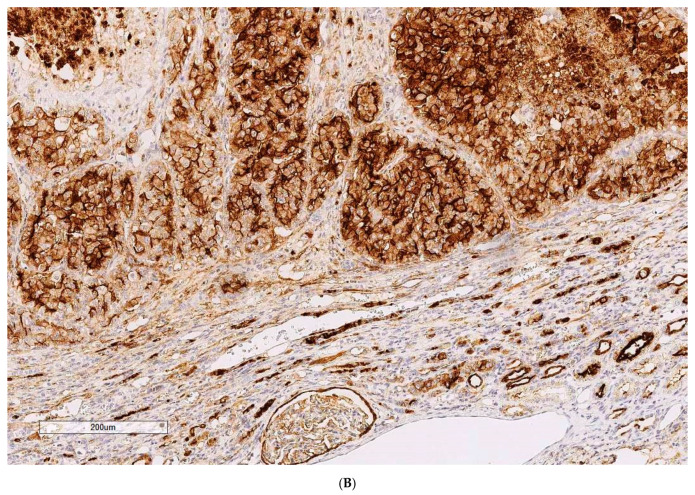
(**A**) Case 1, Vimentin (100×). (**B**) Case 1, CD10 (100×).

In confirming a renal cell origin for rat/OTA tumours, all four cases were diffusely and intensely positive for vimentin (Figure 1A, Case 1, tumour dimensions 10 × 20 mm)

For case 1, CD10 is diffusely and intensely positive, as illustrated on approximately the same area as vimentin, in Figure 1B. 

**Figure 2:**
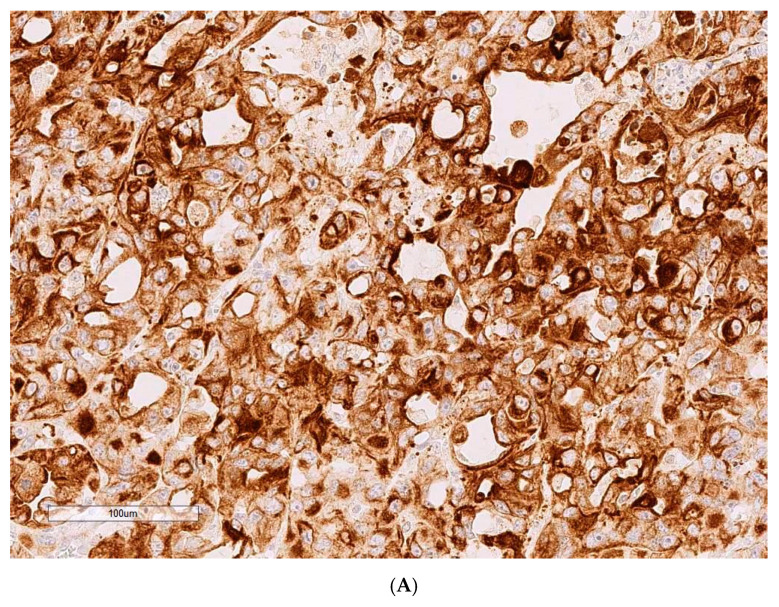

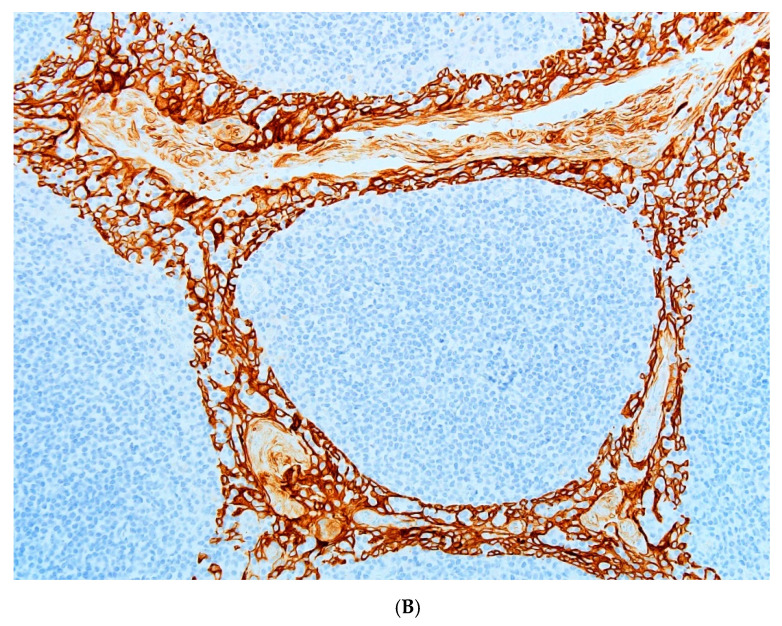
(**A**) Case 2, CK MNF 116 (200×). (**B**) Control (human tonsil), CK MNF 116 (200×).

For Case 2, tumour diameter 20 mm, CD10 is negative but cytokeratin clone MNF 116 is diffusely positive, with heterogeneous pattern and variable intensity (Figure 2A, with adjacent Figure 2B as positive control illustrating staining in human tonsil and validating immunostaining cross reactivity in rat).

**Figure 3:**
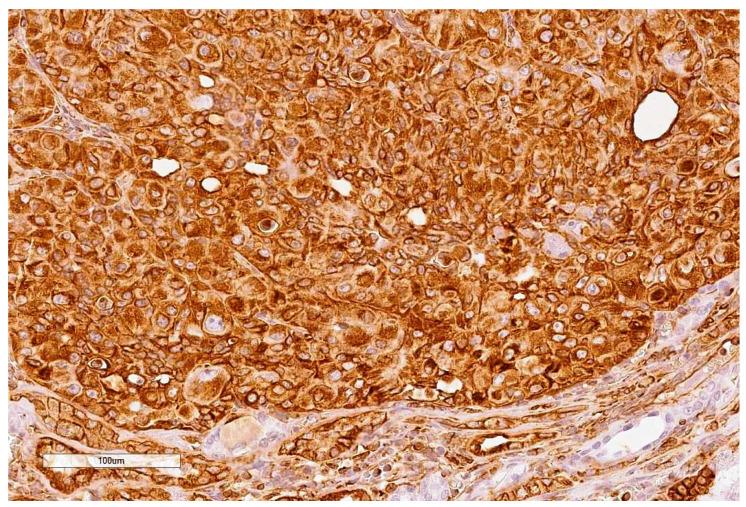
Case 3, Vimentin (200×).

Case 3 tumour (5 mm) is not immuno-positive other than for vimentin (Figure 3); the diagnosis of renal cell carcinoma is being made on the histological aspect on H&E.

Case 4 tumour (5 mm) is additionally positive for CD10. (not illustrated).

## 3. Discussion

Histopathology and immune profiles of the present four rat renal tumours caused by chronic exposure to dietary OTA are complementary to our other similar tumours to which immunohistochemical profiles have recently been ascribed [9]. The combined profiles in ten cases dis-associate the rat tumours from implying a model for an etiological role of OTA for the renal pelvic tumours sometimes associated with the Balkan endemic nephropathy. The latter have also recently been shown to have an immune profile indistinguishable from urothelial tumours studied in Slovakia where the Balkan nephropathy has not been reported, thereby dis-associating OTA from urothelial tumours sometimes occurring in Balkan nephropathy cases. A putative model role for human renal cell cancer might still persist if the vital factor of extraordinary dietary exposure to OTA could be established at a plausibly indicative value. Suggestion of OTA involvement in human testicular cancer has also been discounted experimentally in rats.

Immunostaining for vimentin was invariably extensive as is typical for human tissues of mesenchymal origin. The accompanying positivity for CD10 in three of the 10 rat tumours could fit for humans [10]. MNF 116 positivity seems to be a useful characteristic for some rat renal tumours but does not feature for human renal cancer [10]. Immunoprofiles for these renal tumours do not fit easily with the OTA/rat renal expression assisting in predicting OTA as a human carcinogen. Notably, a recent review, co-authored from IARC [11], concludes that defining DNA adducts, oxidative stress and epigenetic factors that operate in humans could lead to reclassification of OTA as a carcinogen. It is not clear whether this indicates official IARC policy. It is unfortunate that authors mis-cite that ‘DNA diploidy in OTA-induced rat tumours is associated to genetic change’ [12]. It is hoped that any further consideration by IARC would consider that much or most of the in vitro toxicology literature avoids relating the OTA concentration, used to obtain a measured result, to how this relates to kidney parenchyma in vivo during carcinogenesis. It is also important to avoid some literature’s assumption of accumulation of OTA in kidney [13].

Gender specificity in rodents is a neglected problem for matching a potential model with the human for whom there seems none for renal cell carcinoma. For the mouse, OTA renal cancer seems specifically male [14] and for the rat is nearly so [2]. A possible explanation for the rat [15] concerns ability of OTA to bind not only to plasma proteins but also to small male-specific urinary peptides in blood. These are subsequently transported through glomerular filtration to pass down nephrons. They follow the usual fate of at least partial salvage absorption in kidney cortex, thereby potentially augmenting delivery of OTA to cortical epithelia. It is hypothesised that the phenylalanine moiety of free OTA predisposes some direct cortical salvage for that essential amino acid in both genders, but that binding to male urinary peptides boosts the overall toxic impact in the potential tissue target for tumorigenesis. Further experimental study could readily detect by analytical gel electrophoresis the extent to which OTA binds to particular urinary proteins, traces of which must escape cortical salvage to appear in urine as pheromones. Such escape is essential in mouse biology, providing the olfactory language of gender and sexuality in the dark. Similarly, in the rat, verification of association of OTA with urinary protein [15] and extension, possibly by use of OTA radiolabelled to high specific activity, could help to clarify some mystery about OTA circulation in blood. OTA binds to serum albumin rather strongly in humans, but the competition in mice and rats between albumin and the smaller proteins that pass though glomeruli is unclear. Some preliminary exploration across puberty in male rats has occurred [16] but there is room for much more in connection with the pharmacokinetics of OTA. Extension of the previous findings [14] was only prevented by the tragic death of the key scientist.

Another way of gaining understanding of the relative dynamics of OTA in blood of male and female rats, to focus on gender differential in renal tumorigenesis, would be by first establishing and quantifying stable circulating OTA in male and female rats during continuous dietary exposure; then to castrate some of the males while continuing the OTA. Predictably, females would have initially attained a higher stable concentration of OTA in blood than males, which were actively excreting more bound to small urinary proteins. Post-castration OTA concentration in male blood could be expected to rise as testosterone-regulated urinary protein synthesis in liver declines. This could be a step towards evaluating the role of urinary proteins in the male rat tendency to develop renal tumours in response to chronic exposure to dietary OTA. 

An experimental challenge to explore could be the use of an established technique of rat renal transplantation [17] to study male kidney neoplasia in a female body exposed to dietary OTA for 9–10 months, and vice versa. The graft would have to remain functional for well over a year. However, it is a curious thought that the global concern for OTA as a risk to human health, and the vast expense of analysis and legislation, could be an illusion created experimentally before discovery that small urinary proteins are sex pheromones in mice and rats, where they may transport OTA into kidney.

The above histopathology review [9] also notes that the very small group of female renal tumours in the 1989 NTP study had rather more variable histopathology than males, but the largest example was found to conform to the same immune profile as for males. Qualitative rat renal tumour pathology in response to OTA has thus been found to have a consistent characteristic pattern across genders.

There had been no agreed conclusion in an EU project (European Commission 2001–2004) concerning putative genotoxic, epigenetic or oxidative stress mechanisms in male rat renal carcinogenesis caused experimentally by OTA. However, definitive structural evidence of DNA adduction was subsequently obtained [18] after MS analysis of a synthetic adduct following preparative isolation from photoreaction of OTA and DNA. A previous plan, for MS data of the principal adduct isolated from kidneys of rats given OTA, had failed only due to misunderstanding that all of the extremely small amount of sample would need to go on the MS probe. Unfortunately, this was not practically a repeatable enterprise.

A third lifetime rat study, in Hannover Germany in the 1990s using Dark Agouti rats with gavage exposure to OTA [19] similar to that of the NTP study, also revealed some renal tumours. However, these were noted mainly as indicators of carcinogenicity in six of 15 individuals whose kidneys were subsequently found to contain OTA/DNA adducts. OTA dose was similar in cumulative amount to that in the high dose of the NTP study [2]. Giving OTA in aqueous vehicle, instead of corn oil, would have caused daily surges in circulating toxin concentration with greater toxic impact. Nevertheless, finding consistent occurrence of adducts in elderly rats correlated with continuing exposure to OTA is several months too late to imply an epidemiological role in tumour initiation. The same applies in principle also to human kidney cancer.

Subsequent use also of male Dark Agouti rats [7] generally corroborated the tumour findings in Hannover. However, this was only by doubling the OTA dose through 9 months of dietary exposure in the first year of life, during which OTA was consumed normally and slowly mainly by rat habit during the 12 h human night period. Other gavage regimens have delivered immediately during human daytime. Predictably, no adducts could have been found a year later due to repair of any formed during OTA exposure. In any case, it would be necessary to perfuse-wash tumorous kidneys in situ to remove vascular blood, within which adducts also occur [20], to demonstrate OTA/DNA adducts within kidney or tumour parenchyma. A similar strategy has also been necessary to explain a misconception of accumulation of OTA in mammalian kidney [13].

Across the three main centres for lifetime experiments, there was a consistently lower incidence of renal neoplasms in females than in males for Fischer, Dark Agouti and Lewis rats. A putative mechanism for this differentiation has been proposed [15]. It requires further experimental verification but can not be applied to humans which do not have analogous urinary proteins. This gender focus for OTA also potentially diminishes rat carcinogenicity relevance to humans and is an open question for other mammals.

In recent years ethical considerations have limited some whole animal experimentation, in addition to the economic cost of experimental mycotoxins, and to the lifetime maintenance cost for rodents and particularly for primates. Thus, the scientific literature has reports mainly on tissue culture experiments. Whereas this may provide model information applicable to some mammalian tissues, there is less confidence in application to kidney with its exceptionally complex internal dynamics for sequential stages in filtration, key metabolite recovery, water and ion regulation, metabolic waste excretion and the huge replication of separate nephrons. Particularly, relative roles of trans-membrane ion transfer of OTA, as a phenylalanine derivative, from capillaries to nephron epithelia versus classical understanding of glomerular filtration, are not clear. Very rare finding of very simple early nuclear proliferation in situ within a nephron is tantalising evidence of early neoplasm. We have not yet seen this for OTA, but are aware of a possible model illustrated in a rat in an experiment on chronic exposure to aristolochic acid [21]. In our experience, continuous dietary exposure of male rats to OTA for 6 months, during which nephron epithelia experience millions of OTA molecules, is insufficient to initiate any renal tumour. Nine or ten months is sufficient, but the subsequent point at which pre-cancerous neoplasms might be visible in serial-sectioned kidney is unknown. Is this largely a matter of statistical probability of causing a critical, highly focal, genetic lesion? More experimental understanding is needed for this topic. OTA exposure starting at one year has failed to cause cancers [22]. What factor(s) between 6 and 9 months of age is influential in OTA tumorigenesis? 

Experimental evidence shows that the Fischer and other OTA/rat tumours which have generated concern for humans may simply mimic the mechanism operating spontaneously in the EKER strain [23]. The findings are reminiscent of constitutive changes in the rat tuberous sclerosis gene complex which in the EKER strain are correlated with renal neoplasms. Thus, rat renal carcinogenesis caused by OTA does not obviously mimic human urinary tract tumorigenesis.

Another tumorigenesis topic in the literature in the past decade concerns whether OTA is a cause of human testis cancer. This was reiterated prominently in a review abstract [24] with the assertion ‘that OTA is a biologically plausible cause of testicular cancer in man’. This was unfortunately based partly on misreading of the literature [22]. There was also insistence that experimental creation of OTA/DNA adducts in testes of newborn mice, from the mother’s intrauterine exposure to a quite large OTA insult (2.5 mg/kg b wt) about 4 days previously, without discounting OTA/DNA adducts in newborn blood. Application of immunohistochemistry to histology review of rat testis tumours has since showed [25] the distinctive difference between the natural rat Leydig cell tumours and the germinal cell preponderance of human testicular tumours.

## 4. Conclusions

Rat and mouse renal tumour response to long-term dietary OTA has cautioned possible analogous cancer risk for humans, but there is yet no verified case of disease. A recent EFSA report [26] expresses continued uncertainty about human risks for OTA contaminations in food. Tumorigenic mechanisms have been proposed from in vitro studies but are unconvincing for tumours of highly focal origin in rodent or human. Review of rat tumour histopathology, including immune profiles, makes the rat a poor model in humans for cancers in kidney and testis. However, several experimental findings for kidney point to OTA mimicking the natural spontaneous renal tumours of the EKER strain. Natural OTA exposure for humans is very much less than that often applied to experimental cells and whole animal carcinogenicity follows a classical log dose/response relationship. Focus on satisfying Bradford Hill criteria for epidemiology of OTA is encouraged to avoid biases.

## 5. Materials and Methods

Four renal tumours from Fischer male rats given protracted dietary OTA (300 µg/kg body weight daily) [6] were embedded in paraffin blocks. Animals were from the same lifetime experimental group [6] as those whose immunoprofiles were previously described [9]. Ethical review and approval were waived for this study because no new live animals were involved. Sections (3 μm) were mounted on charged slides (TOMO, Matsunami, Japan) and processed for immunohistochemistry in the Cell Pathology Laboratory of South West London Pathology at St George’s Hospital, Tooting, variously applying panels of antibodies in fully automated BenchMark ULTRA immunohistochemistry processing, as required to assist clinical diagnoses. Procedures followed exactly those previously described [9]. The following antibodies were used: CK MNF 116, clone MNF 116 (Dako); Vimentin, clone V9 (Dako, Novocastra); CD10, clone 56C6 (Dako). After applying DAB chromogen, nuclei were counterstained blue with haematoxylin. The brown immune reaction product of DAB chromogen is cytoplasmic and/or membranar for the listed antibodies. Haematoxylin and Eosin staining was also performed for preliminary standard tissue differentiation of nuclear (blue) and cytoplasmic components (red).
